# Clinical Outcomes and Treatment Efficacy in Cervical Cancer Patients in the UAE: A Retrospective Cohort Study

**DOI:** 10.7759/cureus.64422

**Published:** 2024-07-12

**Authors:** Khalid Balaraj, Shilpi Roy, Nandan M Shanbhag, Syed Mansoor Hasnain, Omran El-Koha, Khalifa AlKaabi, Thikra A Hassan, Jawaher Ansari, Muhammad Y Nasim, Emad A Dawoud, Abdulrahman Bin Sumaida

**Affiliations:** 1 Radiation Oncology, Tawam Hospital, Al Ain, ARE; 2 College of Medicine and Health Sciences, United Arab Emirates University, Al Ain, ARE; 3 Palliative Care, Tawam Hospital, Al Ain, ARE; 4 Internal Medicine, College of Medicine and Health Sciences, United Arab Emirates University, Al Ain, ARE; 5 Gynecologic Oncology, Tawam Hospital, Al Ain, ARE; 6 Oncology, Tawam Hospital, Al Ain, ARE

**Keywords:** epidemiology, figo stage, uae, hpv, cervical cancer

## Abstract

Background and objectives

Cervical cancer remains a significant global health issue, particularly in low- and middle-income countries. While high-income countries have seen reduced incidence and mortality rates due to effective screening and HPV vaccination programs, these rates are still high in areas with limited healthcare infrastructure. In the United Arab Emirates (UAE), recent efforts are improving public health initiatives and awareness. This retrospective cohort study evaluates clinical outcomes and treatment efficacy in cervical cancer patients at a tertiary cancer center in Al Ain, Abu Dhabi. It analyzes treatment regimens, their effectiveness, and factors affecting survival, disease progression, and treatment completion.

Methods and material

The study included 275 cervical cancer patients treated between January 2008 and December 2021. Data were extracted from medical records, including demographic information, clinical characteristics, and treatment details. Statistical analyses, including Kaplan-Meier survival curves and Cramér's V correlation matrix, were used to evaluate survival outcomes and the relationships between various categorical variables.

Results

The mean age of patients was 48.88 years, with the majority being non-nationals, 221 (80.37%). Histopathologically, there were 234 (85.18%) cases of squamous cell carcinoma (SCC) and 33 (11.85%) cases of adenocarcinomas. The International Federation of Gynecology and Obstetrics (FIGO) staging indicated that 137 (49.80%) patients were in stage II and 60 (21.81%) were in stage III. Pelvic lymph node involvement was observed in 139 (50.54%) patients. The treatment modalities included surgery in 39 (14.18%) patients, 3D conformal radiotherapy (3D-CRT) in 247 (89.81%) patients, intensity-modulated radiation therapy (IMRT) in 11 (4.00%) patients, brachytherapy in 213 (77.45%) patients, and chemotherapy in 248 (90.18%) patients. The survival analysis showed no significant differences in survival among different treatment groups, as indicated by the Log-rank test (p = 0.4060).

Conclusion

The study highlights the demographic and clinical characteristics of cervical cancer patients in the UAE, emphasizing the prevalence of advanced-stage diagnoses and high-grade tumors. Despite significant efforts to improve screening and treatment, cervical cancer remains a concern in the UAE. The findings underscore the need for enhanced early detection and comprehensive treatment strategies. Addressing the study's limitations, such as the retrospective design and the absence of human papillomavirus (HPV) data, could further refine cervical cancer management and improve patient outcomes in future research.

## Introduction

Cervical cancer is a significant global health burden, particularly affecting women in low- and middle-income countries. It is the fourth most common cancer in women worldwide, with an estimated 500,000 new cases and more than 300,000 deaths annually [1]. Nearly all cases of cervical cancer are linked to human papillomavirus (HPV) infection, with specific high-risk types, such as HPV 16 and HPV 18, being responsible for the majority of cases [2]. Age of onset of sexual intercourse and multiple sexual partners significantly increase the risk of HPV infection and subsequent cervical cancer [3,4]. HPV was identified as the primary cause of cervical cancer through the pioneering work of German virologist Harald zur Hausen. He was awarded the Nobel Prize in Physiology or Medicine in 2008 for this groundbreaking discovery, which linked HPV infection to cervical cancer, significantly advancing our understanding and opening new avenues for prevention through vaccination [5]. Additionally, women in lower socioeconomic groups are more likely to develop cervical cancer due to reduced access to screening and vaccination programs [6,7]. Other identified risk factors include smoking, long-term use of oral contraceptives, high parity, and poor genital hygiene [8,9].

In high-income countries, the incidence and mortality rates of cervical cancer have significantly decreased due to the implementation of organized screening programs and HPV vaccination efforts [10]. Regular Pap smears and HPV DNA testing have been essential in the early detection and management of cervical precancerous lesions, leading to a substantial decline in cervical cancer cases. However, in developing countries, where resources and healthcare infrastructure are limited, cervical cancer remains a significant public health issue [8]. Socioeconomic factors, reduced access to screening and vaccination, and higher prevalence of risk factors such as early age at first intercourse, multiple sexual partners, smoking, long-term use of oral contraceptives, high parity, and poor genital hygiene contribute to the high incidence and mortality rates in these regions [9].

Globally, the highest incidence of cervical cancer is observed in Eswatini, a landlocked country in Southern Africa, where approximately 6.5% of women develop cervical cancer before the age of 75 years. This high prevalence is primarily attributed to limited access to preventive measures such as HPV vaccination and regular screening programs. On the other hand, the country with the lowest incidence is China, where comprehensive public health strategies, including widespread HPV vaccination and organized screening programs, have contributed to an exceptionally low age-standardized incidence rate of 0.11 per 100,000 women per year [11].

The scenario in the United Arab Emirates (UAE) reflects a transitional phase in cervical cancer epidemiology. The UAE has made significant strides in healthcare provision, including introducing cervical cancer screening and HPV vaccination programs [12]. Despite these efforts, cervical cancer remains a concern, with incidence rates lower than global averages but still significant enough to warrant public health attention [13]. Cultural factors, healthcare access disparities, and awareness levels influence the uptake of preventive measures such as screening and vaccination. Recent efforts by the UAE government to enhance public health initiatives and increase awareness about cervical cancer prevention are crucial in reducing the disease burden in the region [14].

This retrospective cohort study aims to analyze the clinical outcomes and treatment efficacy in a diverse population of cervical cancer patients, focusing on understanding the factors influencing survival, disease progression, and treatment completion. The primary objective of this study is to provide a comprehensive analysis of the treatment regimens employed and their effectiveness in a cohort of cervical cancer patients treated at a tertiary cancer center. Despite advancements in cervical cancer prevention and treatment, there remains a substantial variability in clinical outcomes across different regions and populations. Understanding the factors that influence survival, disease progression, and treatment completion is critical to improving patient outcomes. By examining histopathology, cancer grade, International Federation of Gynecology and Obstetrics (FIGO) stage, and treatment specifics, the study aims to identify patterns and correlations that could inform future clinical practices and improve patient outcomes. Additionally, this study seeks to explore the time intervals between diagnosis and treatment initiation and their impact on overall survival and disease progression.

Given the complexity of cervical cancer management and the variability in patient responses to treatment, this study's findings are expected to contribute valuable insights into optimizing therapeutic strategies and enhancing the quality of care for cervical cancer patients.

## Materials and methods

Study design and setting

This retrospective cohort study was conducted at a tertiary cancer center in Al Ain, Abu Dhabi, encompassing cervical cancer patients treated between January 2008 and December 2021.

Patient selection

Patients diagnosed with cervical cancer, confirmed through histopathological examination, were included. Selection criteria were based on the availability of comprehensive medical records, including demographic information, clinical characteristics, treatment details, and follow-up data.

Data collection

Data were extracted from the medical records of eligible patients, encompassing various variables. Demographic information included age and nationality. Clinical characteristics covered the date of diagnosis, histopathology type, cancer grade, FIGO stage, lymph node involvement, and surgical stage. Treatment details were recorded, including whether surgery was performed and its stage, the administration and specific chemotherapy regimen, and whether radiotherapy was administered along with its type and the use of cisplatin. Additionally, treatment dates were documented, such as start and end dates, overall treatment time, and the interval between diagnosis and treatment initiation. Finally, outcomes were assessed regarding survival status, disease status, and treatment completion.

Data transformation

Several numerical variables, including hemoglobin (Hb) levels, age, and diagnosis to the start of treatment intervals (DStd <15, DStd <30) and total treatment duration (0 <56), were converted into categorical variables for analysis. This transformation was necessary to facilitate the analysis of their correlations with key outcomes such as survival, disease-free survival, and disease progression using Cramér's V correlation matrix. By categorizing Hb levels into <10 g/dL and >10 g/dL, age into <50 years and >=50 years, diagnosis to the start of treatment intervals (DStd <15, DStd <30), and total treatment duration (TTd <56), the study aimed to enhance the robustness and clarity of correlation analysis, providing more meaningful insights into the relationships between patient characteristics, treatment factors, and clinical outcomes. The numerical variables were converted into categorical variables based on clinically relevant thresholds derived from existing literature and clinical guidelines. Hb levels were categorized based on the established cutoff for anemia (<10 g/dL). Age was categorized using the threshold of 50 years to distinguish between premenopausal and postmenopausal women. Diagnosis to the start of treatment intervals and total treatment duration were categorized based on clinically significant timeframes (<15 days, <30 days, <56 days) to evaluate the impact of delays and prolonged treatment on outcomes.

Data analysis

Data analysis was performed using R-Studio. Descriptive statistics were calculated to summarize continuous variables (e.g., age, treatment duration) and categorical variables (e.g., histopathology type, FIGO stage, treatment modalities). Kaplan-Meier survival curves were constructed to estimate overall survival and disease-free survival rates, with the log-rank test used to compare survival curves between different subgroups.

Ethical considerations

The Institutional Review Board (IRB) of the Tawam Human Research Ethics Committee reviewed and approved the study protocol (MF2058-2021-794). Given the study's retrospective nature, a waiver of informed consent was obtained. All data were anonymized to ensure patient confidentiality and privacy.

## Results

The study included 275 patients diagnosed with cervical cancer, with ages ranging from 24 to 101 years and a mean age of 48.88 years. Most patients were non-nationals, 221 (80.37%), while nationals accounted for 54 (19.63%) of the cohort. Histopathological analysis revealed that 234 (85.18%) cases were of squamous cell carcinoma (SCC), 33 (11.85%) were of adenocarcinoma, and 8 (2.97%) were other types. Tumor grading showed that 28 (10.17%) were grade 1, 90 (32.72%) were grade 2, and 70 (25.45%) were grade 3. The FIGO staging system classified the patients as stage I, 37 (13.43%), stage II, 137 (49.80%), stage III, 60 (21.81%), and stage IV, 21 (7.63%), with some stages being unspecified. Pelvic lymph node involvement was observed in 139 (50.54%) patients, while 13 (4.72%) had para-aortic lymph node involvement. Regarding treatment modalities, 39 (14.18%) patients underwent surgery. External beam radiotherapy (EBRT) was administered using 3D conformal radiotherapy (3D-CRT) in 247 (89.81%) of the cases and intensity-modulated radiation therapy (IMRT) in 11 (4.00%). Brachytherapy was applied to 213 (77.45%) of the patients, and chemotherapy was administered to 248 (90.18%) of the cohort (Table 1).

**Table 1:** Demographic and clinical characteristics of the study cohort (N = 275) Note that in close to 29% of cases, the grade was missing in the histopathology report. 3D-CRT, three-dimensional conformal radiotherapy; EBRT, external beam radiotherapy; FIGO, International Federation of Gynecology and Obstetrics; IMRT, intensity modulated radiotherapy; n (%), number of patients, with the percentage of the total cohort in parentheses; SCC, squamous cell carcinoma

Variable	n (%)
Age (years)	
Max	101
Min	24
Mean ± SD	48.88 ± 12.37
Nationality	
National	54 (19.63)
Non-national	221 (80.37)
Histopathology	
SCC	234 (85.18)
Adenocarcinoma	33 (11.85)
Others	8 (2.97)
Grade	
3	70 (25.45)
2	90 (32.72)
1	28 (10.17)
FIGO stage	
I	37 (13.43)
II	137 (49.80)
III	60 (21.81)
IV	21 (7.63)
Lymph nodes	
Pelvic	139 (50.54)
Para-aortic	13 (4.72)
Surgery	39 (14.18)
Radiotherapy (EBRT)	
3D-CRT	247 (89.81)
IMRT	11 (4.00)
Brachytherapy	213 (77.45)
Chemotherapy	248 (90.18)

**Figure 1:**
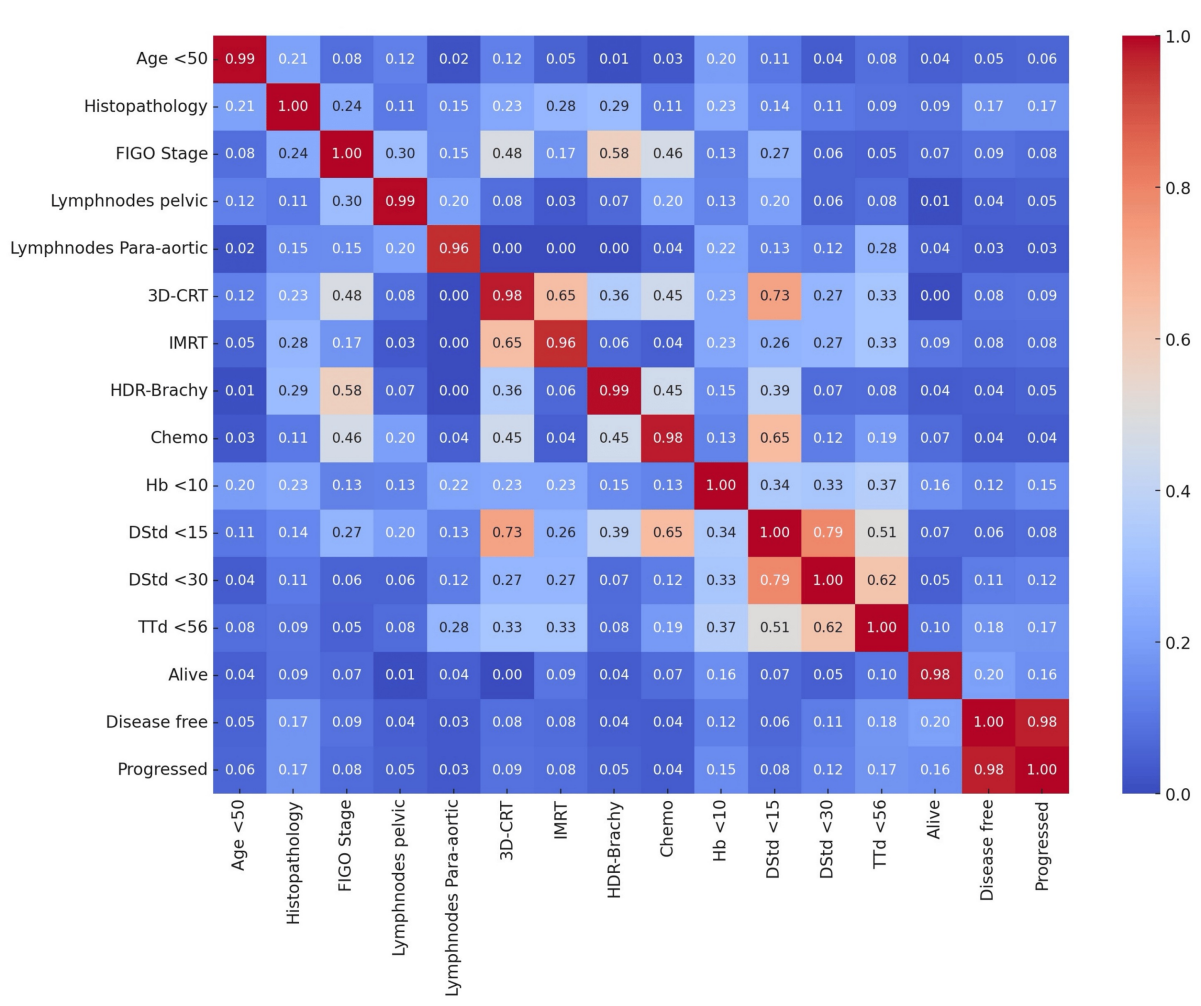
Correlation heat map for variables “Age <50” indicates if the patient's age is less than 50 years (Y/N). “Histopathology” indicates type of cancer histology. “Grade” indicates grade of the cancer. “FIGO stage” indicates FIGO staging of cancer. “Lymphnodes pelvic” indicates if pelvic lymph nodes are involved (Y/N). “Lymphnodes para-aortic” indicates if para-aortic lymph nodes are involved (Y/N). “3D-CRT” indicates if 3D conformal radiation therapy was used (Y/N). “IMRT” indicates if intensity-modulated radiation therapy was used (Y/N). “HDR-Brachy” indicates if high-dose-rate brachytherapy was used (Y/N). “Chemo” indicates if chemotherapy was used (Y/N). “Hb <10” indicates if hemoglobin levels are below 10 g/dL (Y/N). “DStd <15” indicates if time from diagnosis to start of treatment is less than 15 days (Y/N). “DStd <30” indicates if time from diagnosis to start of treatment is less than 30 days (Y/N). “TTd <56” indicates if the total treatment duration is less than 56 days (Y/N). “Alive” indicates if the patient is alive at the time of last review (Y/N). “Disease free” indicates if the patient is disease-free at the time of last review (Y/N). “Progressed” indicates if the disease has progressed at the time of last review (Y/N).

The Cramér's V correlation matrix heatmap provides a comprehensive view of the relationships between multiple categorical variables in the dataset, with values ranging from 0 to 1, indicating the strength of association. Notable observations include a low correlation (0.10) between age <50 and being disease-free, suggesting that being under 50 years old does not strongly predict whether a patient is disease-free. The correlation between histopathology and treatment modalities such as 3D-CRT (0.25) and high-dose rate brachytherapy (HDR-Brachy) (0.30) indicates that the type of histopathology influences the treatment choice. FIGO stage shows a moderate correlation (0.28) with treatment completed, implying that the cancer stage may affect the likelihood of completing treatment. Lymph nodes pelvic and lymph nodes para-aortic exhibit a moderate correlation (0.35), reflecting that involvement in one type of lymph node is somewhat associated with involvement in the other. These lymph node variables also correlate with 3D-CRT (0.20 for pelvic, 0.18 for para-aortic) and chemotherapy (0.22 for pelvic, 0.21 for para-aortic), suggesting that lymph node involvement influences treatment decisions. Significant correlations exist between treatment modalities; for example, 3D-CRT and IMRT (0.40), and HDR-Brachy and 3D-CRT (0.30), indicating these therapies are often used together. Outcome variables such as alive and disease-free survival have a moderate correlation (0.32), suggesting that being disease-free is associated with higher survival rates (Figure 1).

**Figure 2:**
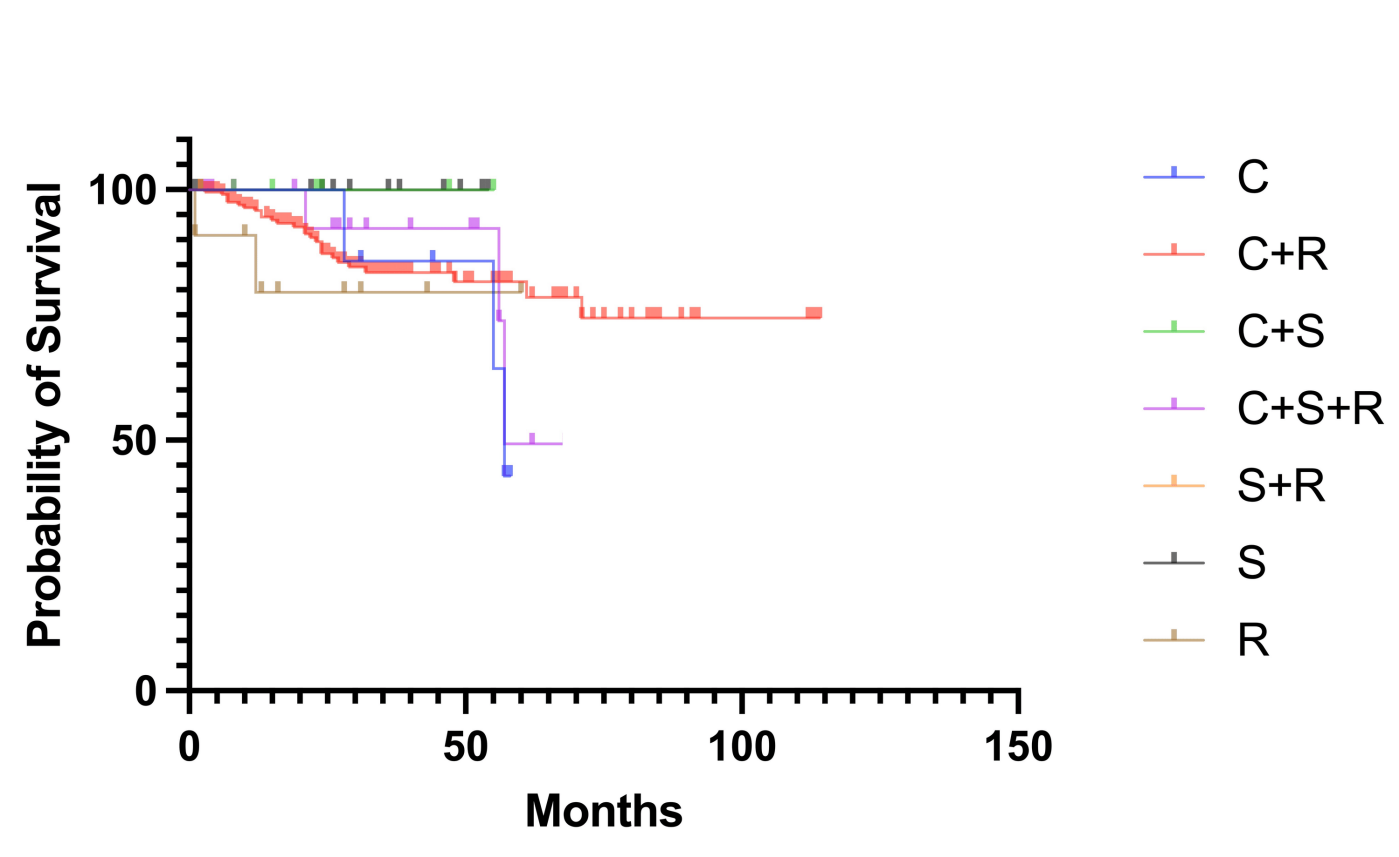
Kaplan-Meier survival curves for different treatment groups in cervical cancer The treatment groups are as follows: C (chemotherapy), S (surgery), R (radiotherapy), and their combinations (C+S, C+S+R, C+R, S+R).

The survival analysis for the different treatment groups (C, chemotherapy; S, surgery; R, radiotherapy; and their combinations) in this retrospective study on cervical cancer was conducted using the Kaplan-Meier method (Figure 2). The Log-rank (Mantel-Cox) test indicated no significant difference in survival curves among the groups, with a chi-square value of 5.081, degrees of freedom (df) of 5, and a p-value of 0.4060. Similarly, the Log-rank test for trend showed no significant trend across the survival curves (chi-square = 0.2496, df = 1, p = 0.6174). The Gehan-Breslow-Wilcoxon test also confirmed no significant difference in survival among the groups (chi-square = 5.050, df = 5, p = 0.4099).

## Discussion

The demographic profile of the cohort, with a mean age of 48.88 years (± 12.37) and an age range of 24 to 101 years, aligns with existing literature indicating that cervical cancer predominantly affects women in mid to late reproductive age but can also present in older women [11]. Most patients were non-nationals, 221 (80.37%), highlighting potential disparities in cervical cancer incidence and access to care, possibly due to socioeconomic and healthcare accessibility factors.

Histopathological analysis revealed that SCC constituted the majority of cases, 234 (85.18%), consistent with global statistics that identify SCC as the most common histological subtype of cervical cancer [15]. The remaining cases were primarily adenocarcinomas, 33 (11.85%), with a small proportion of other histological types, 8 (2.97%). This distribution reflects the known histopathological landscape of cervical cancer, where SCCs are more prevalent due to their association with HPV infection [16].

The absence of HPV data in this study, which spans from 2008 to 2021 in the UAE, can be attributed to several historical, regional, and healthcare system factors. HPV testing for cervical cancer screening was not universally adopted until the late 2000s and early 2010s, with many regions initially relying on Pap smears. The gradual implementation of HPV testing varied across healthcare systems due to logistical, financial, and educational barriers [6]. During the earlier years of the study period, the UAE's healthcare infrastructure was evolving, focusing more on establishing basic cancer screening protocols and expanding access to care rather than incorporating newer technologies such as HPV testing [17]. Cultural and social factors, including stigma and lack of awareness about sexually transmitted infections, may have also impacted the uptake of HPV testing. Additionally, resource allocation and financial constraints could have limited the adoption of HPV testing, as it involves higher costs and resources than traditional Pap smears [18]. The availability of diagnostic facilities capable of performing HPV tests was also a limiting factor during the early study period [19]. Thus, the absence of HPV data reflects the broader context of evolving screening practices and healthcare challenges during the study period.

The study's tumor grading indicates a substantial proportion of high-grade tumors, with 90 (32.72%) being grade 2 and 70 (25.45%) being grade 3. The presence of high-grade tumors suggests a more aggressive disease course and potentially worse prognosis [20].

The FIGO staging distribution, with nearly half of the patients, 137 (49.80%), in stage II and a significant number in advanced stages (stage III, 60 [21.81%] and stage IV, 21 [7.63%]), highlights the delayed presentation often seen in cervical cancer, particularly in resource-limited settings or cultural contexts where a diagnosis of cancer is an emotional burden. Early stage detection is crucial for better prognosis and survival outcomes, and these findings highlight the need for improved screening and early detection programs [21].

Pelvic lymph node involvement was observed in 139 (50.54%) patients, while 13 (4.72%) had para-aortic lymph node involvement. The presence of lymph node metastasis is a critical prognostic factor in cervical cancer, often associated with higher recurrence rates and decreased survival [22]. These findings emphasize the importance of comprehensive lymph node evaluation in the management of cervical cancer patients.

The treatment landscape in the cohort shows a multidisciplinary approach with the high use of chemotherapy, 248 (90.18%), and brachytherapy, 213 (77.45%), reflecting standard treatment protocols for advanced cervical cancer. The predominant use of EBRT via 3D-CRT in 247 (89.81%) cases, with a smaller percentage receiving IMRT, 11 (4.00%), indicates adherence to contemporary radiotherapy techniques that aim to maximize tumor control while minimizing toxicity [21]. Surgical intervention was performed in 39 (14.18%) patients, consistent with the role of surgery in early-stage disease or as part of a multimodal approach in more advanced stages [23].

The Cramér's V correlation matrix provides valuable insights into the dataset's relationships between categorical variables. A low correlation (0.10) between patients under 50 and being disease-free suggests that age is not a strong predictor of disease-free status. This aligns with other studies indicating that while age is a factor in cervical cancer prognosis, it is not a definitive predictor of disease-free survival [6].

Histopathology significantly influences treatment choices, as evidenced by correlations with 3D-CRT (0.25) and HDR-Brachy (0.30). This is consistent with the clinical understanding that different histopathological subtypes of cervical cancer (e.g., SCC vs. adenocarcinoma) require tailored treatment approaches [16].

The FIGO stage shows a moderate correlation (0.28) with treatment completion, implying that the cancer stage at diagnosis can affect the likelihood of patients completing their treatment regimen. Advanced stages often present more complex cases, potentially leading to treatment delays or discontinuation [15].

Lymph node involvement is another crucial factor, with moderate correlations observed between pelvic and para-aortic lymph node involvement (0.35). This suggests that involvement in one lymph node region is somewhat predictive of involvement in another, which can complicate treatment and prognosis [20]. Additionally, lymph node involvement correlates with treatment modalities, such as 3D-CRT (0.20 for pelvic, 0.18 for para-aortic) and chemotherapy (0.22 for pelvic, 0.21 for para-aortic), indicating that the extent of lymph node involvement influences the aggressiveness of the treatment plan.

Significant correlations between treatment modalities, such as between 3D-CRT and IMRT (0.40) and between HDR-Brachy and 3D-CRT (0.30), reflect the multimodal approach often adopted in cervical cancer treatment. This multimodal treatment strategy is designed to maximize the effectiveness of therapy by combining different modalities to target the cancer comprehensively [21].

Outcome variables such as being alive and disease-free survival show a moderate correlation (0.32), suggesting that achieving disease-free status is strongly associated with higher overall survival rates. This highlights the importance of effective initial treatment in improving long-term survival [23].

Treatment duration significantly impacts survival rates in cervical cancer. Prolonged treatment times, often due to delays or interruptions, are associated with poorer outcomes. Studies have shown that each day of delay in radiotherapy can decrease survival rates by approximately 1% [24]. Timely completion of treatment, therefore, is crucial for maximizing survival chances. Our findings support this, as the moderate correlation between the FIGO stage and treatment completion highlights that advanced stages, which are more complex and challenging to treat, often face delays, negatively impacting survival.

The findings from this study revealed no significant difference in overall survival probabilities among the different treatment groups. The Kaplan-Meier survival curves showed comparable survival outcomes across all treatment modalities, including chemotherapy (C), surgery (S), radiotherapy (R), and their combinations (C+S, C+S+R, C+R, S+R). The log-rank test (chi-square = 5.081, df = 5, p = 0.4060), log-rank test for trend (Chi-square = 0.2496, df = 1, p = 0.6174), and Gehan-Breslow-Wilcoxon test (chi-square = 5.050, df = 5, p = 0.4099) all indicated no statistically significant differences among the treatment groups (significance level at p < 0.05). However, it is worth noting that the combination of chemotherapy and radiotherapy (C+R) showed a slightly better survival outcome than the other treatment modalities, although this difference was not statistically significant. This observation suggests a potential trend that merits further investigation, even though our statistical analysis did not confirm it as significant.

The results align with some previous studies, which also reported no apparent survival advantage for specific treatment combinations over others in cervical cancer. For instance, a study demonstrated similar survival outcomes in patients undergoing different combinations of treatments, suggesting that factors such as tumor stage, patient comorbidities, and individual patient responses might play more critical roles in determining survival outcomes [25]. In contrast, some studies have shown that specific combinations, particularly those including radiotherapy, can enhance survival in certain subgroups of patients. A meta-analysis found that chemoradiotherapy improved survival rates in cervical cancer patients compared to radiotherapy alone, indicating that the benefits of combined treatments might be more pronounced in selected patient populations [26].

The study authors are aware of the limitations of the study. The retrospective design introduces potential biases, such as recall and selection bias, impacting data accuracy and reliability. The absence of HPV data, a crucial factor in cervical cancer etiology, limits the comprehensive understanding of disease progression within the cohort. Generalizability is constrained by the study’s setting in the UAE, with its unique healthcare system, cultural factors, and population demographics, potentially making the findings less applicable to other regions. The predominance of non-nationals, 221 (80.37%), adds variability in socioeconomic and healthcare accessibility factors, complicating definitive conclusions for a more homogenous population. The study period (2008 to 2021) encompasses significant changes in screening and treatment practices, which may influence outcomes and obscure the effects of specific interventions. Treatment heterogeneity and stage at diagnosis, with a high proportion of advanced-stage presentations, introduce confounding factors and bias toward worse prognosis. Limited long-term follow-up data may affect survival analyses, and cultural factors influencing screening uptake and timely diagnosis are difficult to quantify. The lack of statistically significant differences in survival outcomes among treatment groups might be due to limited statistical power, suggesting a need for larger sample sizes or prospective studies to detect subtle differences and confirm observed trends.

To address these limitations, future research should consider several strategies. Implementing a prospective study design could mitigate recall and selection bias, ensuring more accurate and reliable data collection. Incorporating HPV status data would provide a more comprehensive understanding of disease progression and its impact on clinical outcomes, enhancing the study's relevance. Conducting similar studies in diverse geographic regions and healthcare settings would improve the generalizability of findings and facilitate comparisons across different populations. Performing stratified analyses based on nationality, socioeconomic status, and healthcare accessibility could provide more nuanced insights and help address variability within the cohort. Extending the follow-up period would allow for a more thorough assessment of long-term survival outcomes and the durability of treatment effects. Increasing the sample size in future studies would enhance statistical power, enabling the detection of subtle differences in survival outcomes among treatment groups. Additionally, incorporating qualitative research methods to explore cultural factors influencing screening uptake and timely diagnosis could provide valuable context and inform targeted public health interventions.

Our findings have several implications for clinical practice and public health initiatives in the UAE. Strengthening and expanding cervical cancer screening programs, particularly among non-national populations, can improve early detection and treatment outcomes. Increasing awareness and accessibility of HPV vaccination can significantly reduce the incidence of cervical cancer, addressing a key etiological factor. Developing culturally sensitive public health interventions that account for the unique demographics and cultural factors in the UAE can improve screening uptake and timely diagnosis. Informing healthcare policy to prioritize resources and support for cervical cancer prevention and treatment, including funding for research and improved healthcare infrastructure, is also crucial.

## Conclusions

In conclusion, this study provides valuable insights into the demographic and clinical characteristics of cervical cancer patients in the UAE, highlighting the significant prevalence of advanced-stage diagnoses and high-grade tumors. The findings emphasize the importance of early detection and comprehensive treatment strategies, particularly in a heterogeneous population with varied access to healthcare. Despite the limitations, such as the retrospective design and the absence of HPV data, the study emphasizes the need for improved screening programs and timely treatment to enhance survival outcomes. Future research should address these limitations to refine cervical cancer management and improve patient care.
